# Novel TLR2-binding adjuvant induces enhanced T cell responses and tumor eradication

**DOI:** 10.1186/s40425-018-0455-2

**Published:** 2018-12-12

**Authors:** Gijs G. Zom, Marian M. J. H. P. Willems, Selina Khan, Tetje C. van der Sluis, Jan Willem Kleinovink, Marcel G. M. Camps, Gijsbert A. van der Marel, Dmitri V. Filippov, Cornelis J. M. Melief, Ferry Ossendorp

**Affiliations:** 1 Department of Immunohematology and Blood Transfusion, section Tumorimmunology, Leiden University Medical Center, Leiden, The Netherlands; 2 Leiden Institute of Chemistry, Leiden University, Leiden, The Netherlands; 3 ISA Pharmaceuticals BV, Leiden, The Netherlands

**Keywords:** Toll-like receptor 2, Adjuvant, Cancer vaccine, Peptide vaccination, T cell activation

## Abstract

**Background:**

Ligands for the Toll-like receptor (TLR) family can induce activation of cells of the innate immune system and are widely studied for their potential to enhance adaptive immunity. Conjugation of TLR2-ligand Pam3CSK4 to synthetic long peptides (SLPs) was shown to strongly enhance the induction of antitumor immunity. To further improve cancer vaccination, we have previously shown that the novel TLR2-L Amplivant (AV), a modified Pam3CSK4, potentiates the maturation effects on murine DCs. In the current study, we further assessed the immunological properties of AV.

**Methods:**

Naïve mice were vaccinated with a conjugate of either Pam3CSK4 or AV and an SLP to assess specific T cell priming efficiency in vivo. The potency of AV and Pam3CSK4, either as free compounds or conjugated to different SLPs, to mature murine DCs was compared by stimulating murine dendritic cells overnight followed by ELISA and flow cytometry analysis. Murine tumor experiments were carried out by vaccinating mice carrying established HPV16 E6 and E7-expressing tumors and subsequently analyzing myeloid and lymphoid cells infiltrating the tumor microenvironment. Furthermore, tumor outgrowth after vaccination was monitored to enable comparison of the efficiency to induce antitumor immunity by Pam3CSK-SLP and AV-SLP conjugates. To enhance therapeutic efficacy, AV-SLP conjugate vaccination was combined with ablative therapies to assess whether synergism between such therapies would occur.

**Results:**

SLPs conjugated to AV induce stronger DC maturation, in vivo T cell priming and antitumor immunity compared to conjugates with Pam3CSK4. Interestingly, AV-SLP conjugates modulate the macrophage populations in the tumor microenvironment, correlating with a therapeutic effect in an aggressive murine tumor model. The potency of AV-SLP conjugates in cancer vaccination operates optimally in combination with chemotherapy or photodynamic therapy.

**Conclusion:**

These data allow further optimization of vaccination-based immunotherapy of cancer by use of the improved TLR2-ligand Amplivant.

**Electronic supplementary material:**

The online version of this article (10.1186/s40425-018-0455-2) contains supplementary material, which is available to authorized users.

## Background

Cells of the innate immune system can recognize pathogen-associated molecular patterns (PAMPs). These specific structures include virus-derived RNA or DNA, bacterial cell wall-derived lipoproteins or sugar groups, amongst others. The receptors of the innate immune system recognizing these structures, pattern-recognition receptors (PRRs), are classified into major receptor families, such as the Toll-like receptor (TLR) family. The latter family consists of 10 receptors in human beings and 13 in mice, which are either expressed on the cell surface or in endosomes of many antigen-presenting cells (APCs) such as dendritic cells (DCs) [1]. Triggering of TLRs by their cognate ligand generally results in activation of APCs and concomitant secretion of pro-inflammatory cytokines and upregulation of co-stimulatory molecules on the cell surface. These effects are essential in the induction of isotype class switching of B cells and induction of efficient T cell responses. Therefore, vaccinologists have extensively explored the use of several TLR-ligands as adjuvants in vaccines to prevent or treat infections and cancer [2–8].

The cell surface-expressed TLR2 can form a heterodimer with either TLR1, TLR6 or in humans also with TLR10. Binding of triacylated lipopeptides to TLR2/1 and binding of diacylated lipopeptides to TLR2/6 leads to pro-inflammatory cytokine release [1], while TLR2/10 heterodimers are thought to act as inhibitory receptors on human B cells and appear not to play a role in DCs [9]. A synthetic triacylated lipopeptide, Pam3CSK4, consisting of a central cysteine residue bound to three palmitic acid chains and a 5 amino acid long peptide (-SK_4_), was shown to be a highly efficient agonist of TLR2 with a favorable solubility profile [10, 11].

In previous studies, we exploited the activating properties of Pam3CSK4 by covalently conjugating the ligand to synthetic long peptides (SLPs) and studied the potential of such constructs as cancer vaccines [12–14]. Not only do Pam3CSK4-SLP conjugates mature APCs, they also have the potential to target conjugated antigens to TLR2-expressing APCs. This was exemplified by the strongly enhanced in vivo T cell priming by Pam3CSK4-SLP conjugates compared to a mixture of free Pam3CSK4 and SLP [14]. Interestingly, while the maturation of DCs by Pam3CSK4 binding was shown to be essential for efficient in vivo T cell priming, TLR2 itself is not the mediator in the uptake of the Pam3CSK4-SLP conjugates [12, 13]. Furthermore, we demonstrated the superiority of Pam3CSK4-SLP conjugates in the induction of antitumor immunity compared to a mixture of free Pam3CSK4 and SLP [14].

Jin et al. co-crystallized the human TLR2/1 heterodimer with Pam3CSK4 and thereby identified the residues of the receptor interacting with Pam3CSK4 [15]. We hypothesized that a hydrogen bond could be formed between the phenylalanine at position 312 of TLR1 and the lipid tail of Pam3CSK4 when a nitrogen atom would be introduced in one of the palmitic acid tails of Pam3CSK4. Indeed, we found that this improved agonist induced enhanced murine DC maturation [16]. We named this novel TLR2-L Amplivant® (AV).

In the present study, we demonstrate the enhanced immunological potency of AV as an adjuvant in cancer immunotherapy. We found that AV conjugated to an SLP induced stronger in vivo T cell priming than Pam3CSK4 conjugated to the same SLP. This may be explained by the enhanced potency to mature APCs by AV, as AV conjugated to several distinct peptide sequences and free AV are consistently superior to Pam3CSK4 in activation of murine DCs. Moreover, anti-tumor vaccination was improved by conjugating AV to an SLP harboring a tumor-specific epitope. Combination treatments of SLP vaccination and either low-dose chemotherapy or photodynamic therapy (PDT) have been described to work synergistically [17, 18]. PDT is based on systemic injection of a photosensitizer, followed by local tumor irradiation with a laser. We show that a combination of these tumor ablative and immunomodulatory therapies and Amplivant-SLP conjugate vaccines also acts synergistically in tumor eradication.

## Methods

### Cell lines

Tumor cell line TC-1 was provided as a kind gift by T.C. Wu (Johns Hopkins University, USA). TC-1 was generated by retrovirally transducing lung fibroblasts derived from C57BL/6 mice with the oncogenic E6 and E7 proteins of HPV16 and c-H-*ras* oncogenes [19]. The TC-1 cells were maintained as previously described [20]. For tumor experiments, cells with a low passage number (< 8) were used. The D1 cell line is a growth factor-dependent murine splenic DC cell line, showing highly similar characteristics to natural occurring DCs [21]. D1 cells were kindly provided by P. Ricciardi-Castagnoli (University of Milano-Bicocca, Italy). The B3Z hybridoma cell line was kindly provided by N. Shastri (University of California, Berkeley, USA) and cultured as described previously [22]. All cell lines were tested by PCR for rodent viruses with negative results. *Mycoplasma* tests routinely performed for all cell lines by PCR were negative.

### Peptides

The following synthetic long peptide sequences were applied in this study, either as free peptide or conjugated to Pam3CSK4 or AV. SLP_OVA CTL_: DEVSGLEQLESIINFEKLAAAAAK, SLP_OVA Th_: ISQAVHAAHAEINEAGR; SLP_HPV_: GQAEPDRAHYNIVTFCCKCDS. Peptides were synthesized and conjugated to AV as described previously [12].

### DC maturation

Bone-marrow derived DCs (BMDC) were isolated from C57BL/6 mouse bone marrow and subsequently cultured for 10 days as described elsewhere [21]. Either BMDC or D1 cells were incubated overnight with the indicated compounds in 96-wells culture plates. Supernatant was taken from the well after incubation, and where indicated cells were harvested and subjected to flow cytometric analysis to determine the expression of co-stimulatory markers.

### Transgenic OT1 T cell activation

The CD8^+^ T cell compartment of OT1 mice fully consists of T cells that are specific for the SIINFEKL CTL epitope of ovalbumin. OT1 mice were sacrificed and spleen and inguinal, brachial and axillary lymph nodes were harvested to obtain OT1 T cells. A single cell suspension was made of the harvested organs using 70 μm strainers (BD Biosciences) and the suspension was subsequently enriched for CD8^+^ T cells using a CD8^+^ T cell enrichment kit (BD). D1 cells pre-loaded for 24 h with the indicated constructs were washed and then co-cultured with the enriched OT1 CD8^+^ T cells (15,000 DC: 50,000 T cells). After 24 h of co-culture, 7.5 *μ*g/ml brefeldin A (Sigma-Aldrich) was added and the cells were left for 16 h at 37 °C/5% CO_2_. Intracellular cytokine staining was performed to stain IFNγ and TNFα in the OT1 CD8^+^ T cells, and subsequent flow cytometry was used to determine the percentage of cytokine-positive OT1 CD8^+^ T cells.

### B3Z hybridoma activation by loading wildtype and TAP^−/−^ BMDC

The B3Z cell line is a hybridoma expressing CD8 and a TCR specific for the SIINFEKL CTL epitope of OVA. Because the sequence of nuclear factor associated with T cell activation (NFAT) is linked to a lacZ reporter construct, a chromogenic substrate conversion can be measured at 595 nm wavelength upon activation of the B3Z cells. Therefore, this T cell hybridoma can be used to measure the level of antigen presentation by APCs independent of co-stimulatory signaling. We loaded 50,000 bone-marrow derived DCs from C57BL/6 mice and TAP^−/−^ C57BL/6 mice per well overnight with the indicated constructs. The next day, the DCs were washed and 50,000 B3Z T cells were added per well for a co-culture at 37 °C. After 24 h, the supernatant was removed and the substrate CPRG (Calbiochem) was added to all wells. A short incubation at 37 °C revealed a color change, measurable at 595 nm wavelength using a microplate absorbance reader (Bio-rad).

### In vivo T cell priming

Naïve C57BL/6 mice (Charles River Laboratories) of 6–8 weeks old were vaccinated subcutaneously in the tailbase with 5 nmole of the indicated ovalbumin-derived constructs dissolved in 50 μl PBS. This dose was selected based on titration experiments conducted in earlier studies, in which we observed strong in vivo T cell induction [14]. Fourteen days later, an identical boost vaccination was given. Five days after the boost, all mice were sacrificed and inguinal lymph nodes and spleen were harvested. A single cell suspension of the organs was made using 70 μm strainers (BD Biosciences). The cells were washed and stained with fluorescent antibodies directed against CD3 and CD8 (eBioscience), tetramers specific for the SIINFEKL CTL epitope, and 7-AAD to exclude dead cells, either directly ex vivo (LN) or after a 7-day restimulation by co-culture with irradiated OVA-expressing EG7 tumor cells (spleen). The stained cells were analyzed by flow cytometry on a FACS Calibur (BD).

Vaccination of C57BL/6 mice with the constructs derived from HPV16 E7 was performed by s.c. vaccination (5 nmole per vaccine) on day 0, 14 and 23. Blood was collected on day 29 and all mice were sacrificed on day 30 to collect spleen and inguinal (draining) lymph node samples. Blood samples were analyzed for the presence of D^b^-RAHYNIVTF tetramer-specific T cells by flow cytometry. A single cell suspension was generated of the spleens and lymph nodes using 70 μm strainers to enable staining with the D^b^-RAHYNIVTF tetramer and antibodies specific for CD3, CD8, CD62L, CD127 and KLRG1 [20]. The stained cells were analyzed by flow cytometry on a FACS Calibur (BD).

### Tumor outgrowth

Naïve C57BL/6 mice (Harlan Laboratories) of 6–8 weeks old were subcutaneously challenged with 100,000 TC-1 ([19]) cells on day 0 in the right flank. On day 8, when tumors were palpable in all mice, or day 10 when indicated, a vaccination with the indicated constructs dissolved in 50 μl PBS was given s.c. in the tailbase. The tumor sizes were measured at least twice a week by use of a caliper. Mice were sacrificed when tumor sizes reached 2000 mm^3^, in accordance with Dutch animal welfare legislation.

### Analysis of tumor-infiltrating lymphocytes and myeloid cells upon vaccination

Naïve C57BL/6 mice (Harlan Laboratories) of 6–8 weeks old were challenged with a TC-1 tumor as described above. On day 10, mice were vaccinated with 5 nmole of the indicated constructs dissolved in 50ul PBS by s.c. tailbase vaccination. Tumor sizes were measured during the course of the experiment by use of a caliper. On day 19, all mice were sacrificed. Approximately 50 ml of sterile PBS was injected intracardially to flush the blood circulation including the tumors. Tumors were accurately dissected from the mice and left in complete medium on ice. The tumors were subsequently cut in small pieces and mashed over 70 μm cell strainers (BD Biosciences) to create a single cell suspension. Cells were washed and stained using fluorescent antibodies (eBioscience) directed against either lymphocyte or myeloid markers. Fc-block using mouse serum was added to the procedure to prevent aspecific antibody binding. Marker expression on cells was analyzed using flow cytometry (LSRII, BD Biosciences). Live myeloid cells were identified as 7-AAD^−^CD3^−^CD11b^+^. Within this gate, several subpopulations were identified as described in the Results section and Fig. 3, based on gating strategies applied by others [23].

### TLR2-L SLP conjugate and cisplatin combined treatment in tumor-bearing mice

Naïve C57BL/6 mice (Harlan Laboratories) of 6–8 weeks old were challenged with a TC-1 tumor as described above. Five nmole of the indicated constructs was injected subcutaneously in the tailbase in 50 μl PBS on day 10, when the average tumor size was 12mm^3^. Cisplatin was injected intraperitoneally in mice of indicated groups on day 10 and 17, at a suboptimal dose of 4 mg/kg as used previously in our group [18]. The tumor sizes were measured 2 or 3 times a week by use of a caliper. Mice were sacrificed when tumor sizes reached 2000 mm^3^, in accordance with Dutch animal welfare legislation.

### Combined TLR2-L SLP conjugate vaccination and PDT treatment in tumor-bearing mice

Naïve C57BL/6 mice (Harlan Laboratories) of 6–8 weeks old were challenged with a TC-1 tumor as described above. For these experiments, 5 nmole of the indicated constructs was injected subcutaneously in the tailbase in 50 μl PBS on day 6 according to optimized protocols in our lab. On day 6, tumors were palpable in all mice. On day 8, photodynamic therapy (PDT) was performed on mice in indicated groups by intravenous injection of 20 mg/kg Bremachlorin photosensitizer per mouse, followed 6 h later by local illumination of the tumor with a 662 nm laser at 116 mW/cm^2^ for 1000 s (total light dose 116 J/cm^2^), as described elsewhere [17]. The tumor sizes were measured 2 or 3 times a week by use of a caliper. Mice were sacrificed when tumor sizes reached 2000 mm^3^, in accordance with Dutch animal welfare legislation.

## Results

### The modified TLR2-L Amplivant improves conjugated SLP vaccination

**Fig. 1:**
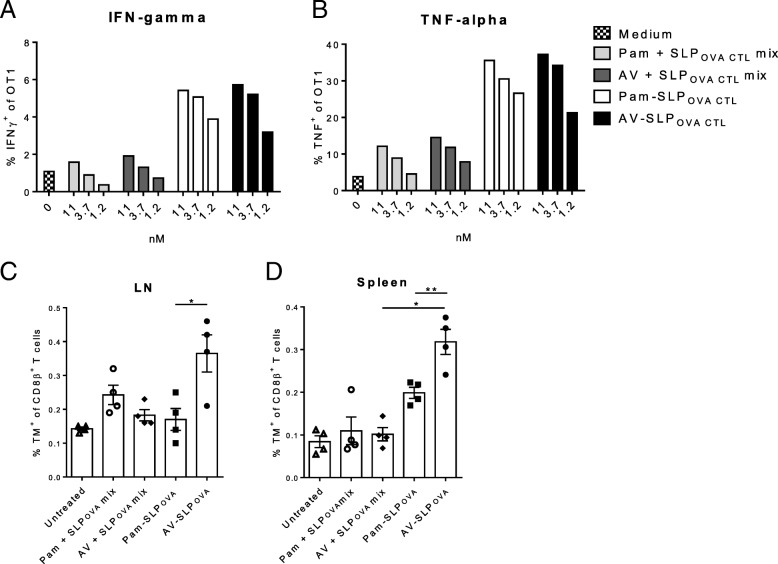
AV-SLP conjugate vaccination induces in vitro T cell activation and enhances in vivo priming of endogenous T cells. Percentages of IFNγ- (**a**) and TNFα- (**b**) producing transgenic OT1 T cells after 48 h of co-culture with D1 DCs that were loaded overnight with the indicated SLP_OVA CTL_ peptide mixed with Pam3CSK4 or AV, or conjugated to Pam3CSK4 or AV. Percentages of K^b^-SIINFEKL^+^ CD8β^+^ T cells in (**c**) vaccine-draining lymph node ex vivo and (**d**) restimulated splenocytes of C57BL/6 mice vaccinated with mixtures of Pam3CSK4 and SLP_OVA CTL_ (Pam + SLP_OVA_ mix), AV and SLP_OVA_ (AV + SLP_OVA_ mix), Pam-SLP_OVA_ conjugate or AV-SLP_OVA_ conjugate. Five nmole of each SLP, free adjuvant or TLR2-L SLP conjugate were administered, i.e. a mixture of TLR2-L and SLP contained 5 nmole of TLR2-L and 5 nmole SLP. Significance determined by unpaired t-test in (**c**) and (**d**): * *p* < 0.05, ** *p* < 0.01. All presented experiments were repeated at least twice with similar outcome

Modification of TLR2-L Pam3CSK4 resulted in the generation of Amplivant (AV), a TLR2-L with enhanced DC activation potency [16]. In previous studies, we have shown that generation of specific T cell responses by SLP is markedly improved by conjugating the SLP to TLR2-L Pam3CSK4 [12–14]. To test whether AV shares the ability to efficiently induce specific T cell responses, we conjugated both AV and Pam3CSK4 to an ovalbumin-derived SLP containing the CD8^+^ T cell epitope SIINFEKL (SLP_OVA CTL_). DCs were loaded with titrating doses of either Pam3CSK4 or AV conjugated to SLP_OVA CTL_ or with mixtures of free SLP and TLR2-L and subsequently co-cultured with SIINFEKL-specific CD8^+^ T cells derived from the transgenic OT1 mouse. At these low concentrations, both Pam3CSK4- and AV-SLP_OVA CTL_ conjugates show a stronger induction of IFNγ and TNFα production by OT1 T cells, as determined by intracellular cytokine staining, than a mixture of free TLR2-L and SLP_OVA CTL_ (Fig. 1a, and b). Processing of the Pam3CSK4- and AV-SLP_OVA CTL_ conjugates occurred with similar efficiency as no difference in OT1 T cell activation was observed. In addition, we observe a total abrogation of MHC class I-dependent activation of a T cell hybridoma (B3Z) specific for the SIINFEKL CTL epitope (Additional file 1: Figure S1) using cells lacking transporter associated with antigen processing (TAP). This indicates that the processing and presentation of the epitope follows a classical cytosolic cross-presentation route after uptake.

Next, we vaccinated groups of naïve C57BL/6 mice subcutaneously (s.c.) with either the Pam3CSK4- or AV-SLP_OVA CTL_ conjugate. All mice received a booster vaccination 14 days later. Seven days after the boost, significantly more K^b^-SIINFEKL tetramer-positive CD8^+^ T cells are detected in the vaccine-draining lymph nodes upon vaccination with the AV-SLP_OVA CTL_ conjugate, compared to Pam3CSK4-conjugate vaccinated mice (Fig. 1c). Also, significantly more specific CD8^+^ T cells are detected in the AV-SLP_OVA CTL_-vaccinated group after ex vivo restimulation of the splenocytes (Fig. 1d). In line with our previous studies, conjugation of a TLR2-L to an SLP improves the induction of specific CD8^+^ T cells as compared to a mixture of free SLP and TLR2-L [14]. As HPV16 is a clinically more relevant tumor antigen, we also conjugated AV and Pam3CSK4 to an SLP derived from HPV16 E7 which harbors a D^b^-restricted MHC class I epitope [24]. After receiving three s.c. vaccinations, an enhanced average percentage of E7-specific CD8^+^ T cells is detected in blood and spleen in the mice vaccinated with AV-SLP_HPV_, albeit not significantly (Additional file 1: Figure S2A, and B). Notably, the mice in the AV-SLP_HPV_ conjugate group with the highest percentage of HPV16 E7-specific CD8^+^ T cells also had a higher percentage of antigen-specific effector/effector memory T cells, as indicated by the low expression of CD127 or CD62L and high expression of KLRG1 (Additional file 1: Figure S2C, and D).

### Amplivant induces potent murine DC activation

**Fig. 2:**
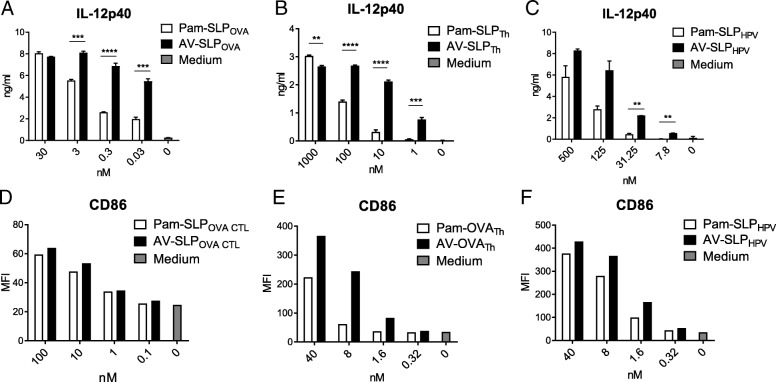
AV retains its superior capacity in induction of DC maturation over Pam3CSK4 upon conjugation. (A – C) Concentration of IL-12p40 in the supernatant of BMDCs stimulated overnight with ovalbumin-derived SLP_OVA CTL_ or SLP_OVA Th_ or HPV16 E7-derived SLP_HPV_, conjugated to either Pam3CSK4 or AV. (D-F) Expression of CD86 (MFI; mean fluorescence intensity) on BMDCs stimulated overnight as in Fig. 2a, B and c. Results are representative of at least three independently performed experiments with each of the conjugated SLPs. Significance determined by unpaired t-test: * *p* < 0.05, ** *p* < 0.01, *** *p* < 0.001, **** *p* < 0.0001

In our previous study, we reported that AV induces enhanced murine DC maturation compared to Pam3CSK4 [16]. Looking more in-depth at the maturation potential of non-conjugated AV reveals enhanced IL-12p40 production and upregulation of MHC class II molecules (I-A^b^) and co-stimulatory markers CD40 and CD86 by both a murine DC cell line and bone marrow-derived DCs (BMDC; Additional file 1: Figure S32) in comparison with non-conjugated Pam3CSK4. To determine whether AV also induces enhanced DC maturation upon conjugation to a an SLP which improved T cell priming, we stimulated murine BMDCs with titrating concentrations of either Pam3CSK4 or AV conjugated to three different SLPs: ovalbumin-derived SLP_OVA CTL_ and SLP_OVA TH_ and HPV16 E7-derived SLP_HPV_ [14, 25]. Indeed, IL-12p40 production by BMDC upon stimulation with AV-conjugated SLP was significantly higher than Pam3CSK4-conjugated SLP (Fig. 2a, b and c). Although not always as pronounced as the IL-12p40 production, also the co-stimulatory marker CD86 was upregulated more strongly in response to stimulation with the AV-SLP conjugate (Fig. 2d, e and f) [24]. A representative dilution range is shown separately for each conjugated SLP as conjugation of AV to an SLP leads to different physicochemical properties for each SLP. Besides, the conditions of each cellular assay are slightly different. These data show that AV retains its effective DC activating capacity upon conjugation to SLPs, irrespective of SLP sequences.

### Favorable modulation of tumor-associated myeloid compartment upon vaccination of tumor-bearing mice with AV-SLP conjugate

**Fig. 3:**
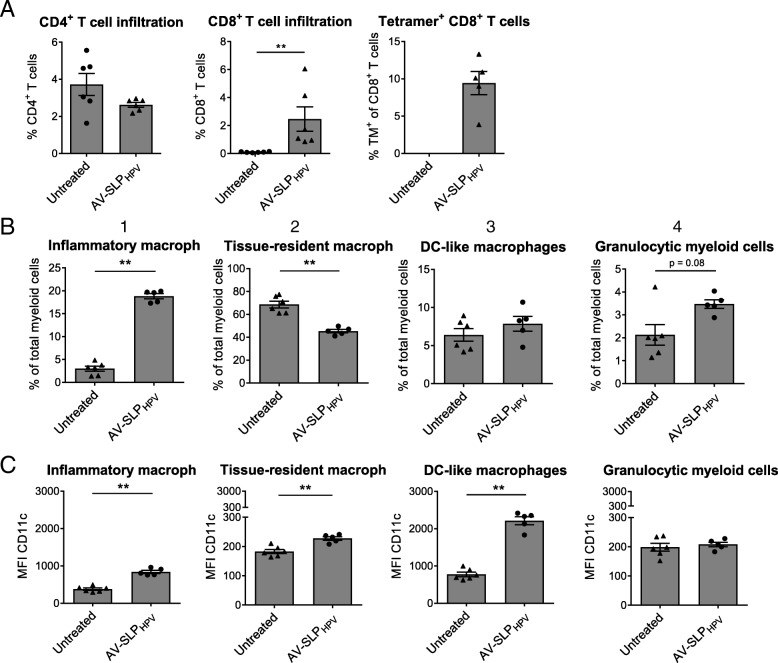
Modulation of intratumoral lymphoid and myeloid compartment by AV-SLP_HPV_ conjugate. Mice were vaccinated ten days after s.c. TC-1 tumor challenge and sacrificed on day 9 after vaccination to analyze tumor-infiltrated lymphocytes and myeloid populations. (**a**) Percentages of CD4^+^ T cells, CD8^+^ T cells (of live cells; 7-AAD^−^) and D^b^-RAHYNIVTF^+^ (HPV16 E7-specific) CD8^+^ T cells in tumor tissue as determined by flow cytometry. One outlier removed from group Pam-SLP_HPV_ in plot of tetramer (TM)^+^ CD8^+^ T cells after performance of Grubb’s test. (**b**) Percentages of described myeloid subpopulations within CD3^−^CD11b^+^ gated live cells from tumor tissue. (**c**) Expression of CD11c on described myeloid subpopulations. Significance determined by unpaired t-test: ** *p* < 0.01

Next, we examined the effects of AV-SLP conjugate vaccination in mice carrying an established tumor. First, naïve C57BL/6 mice were challenged with TC-1 tumor cells which express the HPV16 oncoproteins E6 and E7 [19]. After 10 days, when average tumor sizes reached 12 mm^3^, mice were s.c. vaccinated with either AV-SLP_HPV_ or vehicle only. Nine days after the vaccination, when the expected peak of the T cell response and tipping point in the tumor regression phase coincide, all mice were sacrificed and the phenotypes of the infiltrating lymphocyte and myeloid populations were analyzed. No significant differences were seen in tumor size at this time point (data not shown). Vaccination with the conjugates led to a strong infiltration of CD8^+^ T cells into the tumor while the percentages of CD4^+^ T cells in the tumor were similar to those in untreated mice (Fig. 3a). Of these CD8^+^ T cells, an average of approximately 10 % was specific for the CTL epitope (RAHYNIVTF) embedded in the conjugated vaccine SLP. In a recent study, four tumor-infiltrating myeloid populations were identified based on the expression of CD11b, F4/80 and Ly6C (Additional file 1: Figure S4.A) [23]. Of these, the frequency of inflammatory macrophages (CD3^−^CD11b^+^Ly6C^hi^F4/80^hi^) after peptide vaccination correlated with the infiltration of CD8^+^ T cells. In the present study, we observe a highly significant increase in the frequency of intratumoral inflammatory macrophages upon vaccination with AV-SLP_HPV_. At the same time, vaccination also led to decreased levels of tissue-resident macrophages (CD3^−^CD11b^+^Ly6C^INT^F4/80^INT^; Fig. 3b). In addition, the AV-SLP_HPV_ conjugate induces stronger upregulation of the DC marker CD11c on all gated myeloid populations except for the granulocytic myeloid cells, which may reflect differentiation towards a more DC-like phenotype. A direct comparison between AV-SLP_HPV_ and Pam-SLP_HPV_ conjugate vaccination revealed that both conjugates are highly similar in their potency to induce tumor infiltration by T cells and modulation of the intratumoral myeloid populations (Additional file 1: Figure S4.B, C and D). Overall, these results provide important insights into the effects of TLR2-L SLP conjugate vaccination in TC-1 tumor-bearing mice, showing the favorable skewing effect towards the inflammatory macrophage phenotype by AV-SLP_HPV_ conjugate vaccination.

### AV-SLP conjugate vaccination improves therapeutic antitumor response

**Fig. 4:**
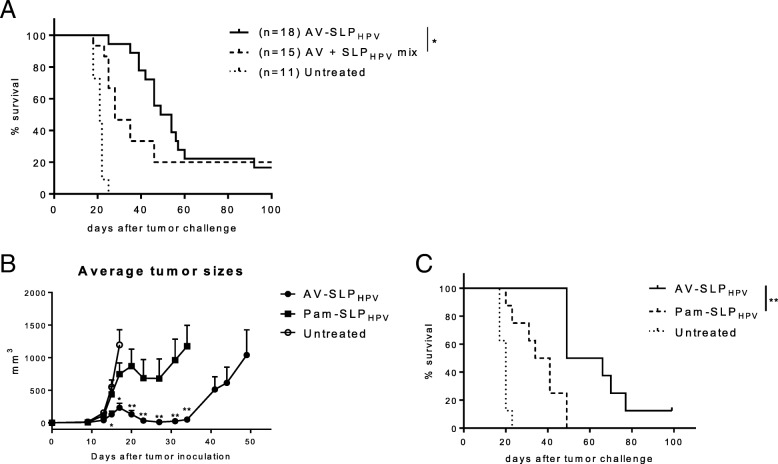
TC-1 tumors initially grow slower in mice vaccinated with AV-SLP_HPV_ conjugate. (**a**) Kaplan-Meier survival plot of C57BL/6 mice carrying a TC-1 tumor vaccinated with indicated vaccines. Data pooled from two identically performed experiments, total number of mice per group indicated as n. Significant difference between AV + SLP_HPV_ mix and AV-SLP_HPV_ (* *p* = 0.014) determined by Gehan-Breslow Wilcoxon test. (**b**) Average tumor size per group of mice vaccinated with either AV-SLP_HPV_ or Pam-SLP_HPV_ conjugates, or not vaccinated (Untreated). Average tumor size followed in time until first mouse was sacrificed due to tumor burden. Significance determined by Mann Whitney t-test (* *p* < 0.05; ** *p* < 0.01). (**c**) Kaplan-Meier plot showing survival of mice vaccinated in same experiment as (**b**). Significance determined by log-rank test (** *p* < 0.01)

In previous studies, we found that conjugates of Pam3CSK4 and SLP were superior to mixtures of Pam3CSK4 and SLP in prophylactic tumor models. In the present study, we s.c. vaccinated mice carrying an established TC-1 tumor with AV-SLP_HPV_ or a mixture of AV and SLP_HPV_ and found that conjugation of AV to SLP_HPV_ significantly enhanced the survival of TC-1-bearing mice in a therapeutic setting (vaccination on day 8 after tumor challenge; Fig. 4a). To study whether the favorable effects in the tumor microenvironment induced by AV-SLP_HPV_ conjugate vaccination led to efficient tumor eradication, C57BL/6 mice carrying a palpable TC-1 tumor were vaccinated with either AV-SLP_HPV_ or Pam-SLP_HPV_. Tumors of mice vaccinated with AV-SLP_HPV_ on day 8 after tumor challenge remained significantly smaller during the initial tumor growth phase than those of mice vaccinated with Pam-SLP_HPV_ (Additional file 1: Figure S5A) which was also reflected in improved early phase survival between day 20 and 40 (Additional file 1: Figure S5B). A stronger and more significant reduction in tumor size was observed when mice were vaccinated with AV-SLP_HPV_ conjugate on day 10 after tumor challenge, when mice have developed larger tumor sizes (approximately 12 mm^3^) at the moment of vaccination (Fig. 4b). Interestingly, the time point at which tumors start to regress in the AV-SLP conjugate-vaccinated group is earlier than in the Pam-SLP conjugate-vaccinated group. Moreover, AV-SLP_HPV_ conjugate vaccination resulted in a significant survival benefit compared with Pam-SLP_HPV_-vaccinated mice (Fig. 4c).

### Combinations of ablative therapy and AV-SLP vaccination synergize in tumor eradication

**Fig. 5:**
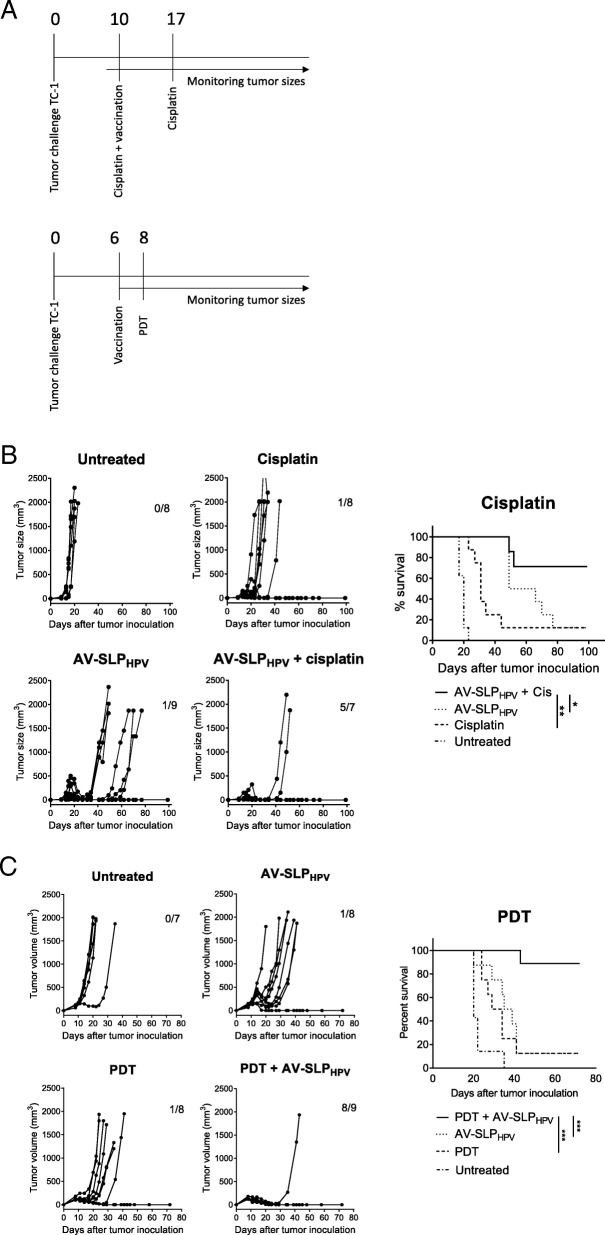
Combination of AV-SLP_HPV_ conjugate vaccination and immunomodulatory therapy in TC-1 tumor model. (A) Overview of experimental setup (timelines). Numbers indicate number of days after tumor challenge. (B) Tumor growth curves and Kaplan-Meier survival plot of combination treatment of AV-SLP_HPV_ conjugate vaccination and i.p. cisplatin treatment. Numbers in graph indicate number of cured mice of total in group. Results are shown of one of two experiments with similar outcome. (C) Combination treatment of AV-SLP_HPV_ conjugate vaccination and photodynamic therapy (PDT). Tumor growth curves and Kaplan-Meier survival plots of groups of mice carrying a TC-1 tumor vaccinated with indicated vaccines. Bottom Kaplan-Meier plot represents survival of mice after re-challenge with TC-1 tumor cells at day 55. Significance determined by log-rank test; * *p* < 0.05; ** *p* < 0.01; *** *p* < 0.001

The observation that only approximately 10–20% of TC-1 tumor-bearing mice are cured by therapeutic vaccination (Fig. 4a, c) shows that AV-SLP conjugate vaccination on its own is not capable to mediate tumor eradication in the majority of animals in this model. In a clinical setting, patients usually receive standard-of-care chemotherapy and/or radiotherapy to treat HPV16-induced cancer. SLP vaccination has recently been shown to synergize with a suboptimal dose of cisplatin in the eradication of TC-1 tumors in mice, partially explained by the enhanced apoptosis of tumor cells by TNFα from SLP-induced T cells in the presence of cisplatin [18]. Therefore, we tested whether synergistic tumor eradication could also be achieved upon combined cisplatin and AV-SLP_HPV_ treatment in TC-1-bearing mice. Mice were vaccinated s.c. with AV-SLP_HPV_ on day 10 after tumor challenge, while cisplatin was injected intraperitoneally at a dose of 4 mg/kg cisplatin, well below the maximum tolerated dose. The treatment timeline is depicted in Fig. 5a. Both cisplatin treatment and AV-SLP_HPV_ vaccination alone delayed the outgrowth of most tumors but were not curative for the majority of mice (Fig. 5b). The combination treatment resulted in a significantly better survival (71% versus 12.5%) compared to the group vaccinated with AV-SLP_HPV_ only.

Another immunomodulatory therapy, photodynamic therapy (PDT), acts by laser-induced local cytotoxicity after administration of the photosensitizer Bremachlorin which accumulates selectively in tumor tissue [17]. We combined PDT with s.c. AV-SLP_HPV_ vaccination to examine whether these treatments act synergistically. PDT treatment or AV-SLP_HPV_ vaccination on itself delayed tumor outgrowth in the TC-1 model (1 mouse out of 8 cured in both groups; 12.5%; Fig. 5c), while the combination of PDT with AV-SLP_HPV_ induced eradication of tumors in 8 out of 9 mice (89%; Fig. 5c). Out of the 8 cured mice, 7 developed a strong memory response as these mice were protected from a rechallenge with TC-1 tumor cells in the opposite flank. Both surviving mice of the AV-SLP_HPV_ and PDT monotherapy groups were protected as well from the rechallenge (Fig. 5c). These data show that AV-SLP conjugate vaccines can be effectively combined with chemotherapy or photodynamic therapy.

## Discussion

We consistently observe enhanced maturation of murine DCs by AV either as a free ligand or conjugated to any peptide sequence tested so far. Interestingly, the physical and chemical properties of each conjugate may differ, as observed before [26]. While the Pam- and AV-SLP_OVA CTL_ conjugates still retain strong activation potency in terms of IL-12p40 induction in the picomolar range, the Pam-SLP_OVA Th_- and Pam-SLP_HPV_-conjugates lose their activity in the low nanomolar range. As the peptide part of each conjugate greatly differs in amino acid composition, also the solubility and binding stability may vary depending on the amount of hydrophilic and hydrophobic residues.

After binding of Pam3CSK4 and AV to TLR2 on the cell surface of DCs and the subsequent maturation process, the constructs are ingested and processed by the DCs resulting in the generation of the exact MHC ligands [12]. Using transgenic OT1 cells as a T cell read-out, we found that both Pam- and AV-conjugated SLPs are efficiently taken up and processed but do not differ in their capacity to induce T cell activation. This may be explained by the fact that the DC activation and concomitant cytokine expression and upregulation of co-stimulatory markers reach a saturating level for activation of the low-threshold OT1 transgenic T cells. Both conjugates outperformed a mixture of SLP and either AV or Pam3CSK4. We have reported the superiority of conjugates over mixtures of TLR2-L and SLP before [12], which can be explained by an enhanced uptake of the TLR2-L SLP_OVA CTL_ conjugates through DC targeting by their lipophilic properties.

In contrast to these in vitro data, a significant difference was found in potency of the two conjugated ligands with respect to T cell priming in vivo, as the AV-SLP_OVA_ conjugates were shown to more effectively expand T cells of the murine endogenous T cell repertoire. Comparing AV-SLP_HPV_ and Pam-SLP_HPV_ conjugate–induced specific T cells, differences in T cell activation markers were less pronounced. However, differential potency in T cell activation by AV and Pam3CSK4 was suggested by skewing of the T cell response towards a more effector (memory) T cell-like phenotype which was observed in part of the mice vaccinated with the AV-SLP_HPV_ conjugate. Based on their general molecular structure Pam3CSK4 and AV share similar in vivo targeting potential. Therefore, the enhanced priming of CD8^+^ T cells is most likely explained by the stronger binding of AV to TLR2-expressing APCs. In a previous study, we have assessed the influence of DC maturation on T cell priming by conjugating either the functional *R*-Pam3CSK4 or dysfunctional *S*-Pam3CSK4 stereoisomer to an SLP [13]. Although *S*-Pam-SLP conjugates could induce T cell priming in vivo, the *R*-Pam-SLP performed significantly better. Therefore, it is likely that the enhanced activation of DCs by AV positively affects the strength of T cell responses.

Given the results discussed above, we analyzed whether AV-SLP conjugates were also more effective as therapeutic vaccines in an established HPV-tumor model. As the data in Fig. 4b and c demonstrate, AV-SLP_HPV_ vaccination has superior antitumor efficacy over Pam-SLP_HPV_ vaccination. In this setting, mice had large established tumors, indicating the strength of AV-SLP_HPV_-induced antitumor immune responses. Mice carrying a tumor that was just palpable (day 8 after tumor challenge) also benefited stronger from AV-SLP_HPV_ than Pam-SLP_HPV_ vaccination, although differences were smaller (Additional file 1: Figure S5) suggesting that antigenic load may affect therapeutic vaccination potency. These data show that the enhanced TLR potency of AV is also beneficial for therapeutic antitumor efficacy.

The mechanism behind the decreased tumor growth in AV-SLP_HPV_-vaccinated mice was studied further by phenotyping the tumor-infiltrating lymphocytes and myeloid cells. The myeloid infiltrate can be highly heterogeneous within tumors but much can be learned from studies focusing on the roles of specific myeloid subtypes in immunization and infection settings [27]. Ly6C^hi^ monocytes are described to be precursors of tumor-associated macrophages and inflammatory DCs. The latter are capable of efficient antigen presentation and retain Ly6C^hi^ expression after differentiation. The Ly6C^hi^ DCs play an important role in the onset of T_H_1 T cell responses and may therefore be considered reminiscent of M1 myeloid cells [28]. The inflammatory macrophages detected in this study also expressed intermediate levels of CD11c (Fig. 3d), the expression of which was strongly upregulated by AV-SLP_HPV_, but it remains to be determined whether these cells share the same fate and properties with the inflammatory DCs.

The expression of TLR2 is very low or absent on T cells but abundant on several myeloid cells [29], which suggests that AV-SLP_HPV_ may have a direct effect on myeloid populations in the tumor area besides the induction of specific T cells via APCs in lymphoid organs. Unfortunately, not much is known about TLR2 expression on these cell populations. Both AV- and Pam-SLP_HPV_ conjugates reduced the frequency of tissue-resident macrophages. Although likely, we cannot conclude whether tissue-resident macrophages have been modulated towards an inflammatory macrophage phenotype. Another theoretical explanation for the observed skewing may be found in the induction of tumor cell death. Ma et al report on the attraction of myeloid cells into the tumor by ATP released from dying cancer cells [30]. These cells could further differentiate towards CD11b^+^CD11c^+^Ly6C^hi^ cells, similar to the inflammatory macrophage population we find in tumors in the current study. Therefore, by directly inducing tumor cell death, AV may indirectly attract and cause differentiation of myeloid populations in the tumor microenvironment. Another attractive explanation is that the tumor microenvironment is influenced by the induced T cell response. As we have shown that AV conjugated to HPV16 E6-derived SLPs can stimulate Th1-type T cells in tumor draining LN cells derived from cervical cancer patients [26], it is conceivable that this type of T cells can indirectly skew the tumor environment to a more favorable myeloid cell composition [23].

Figure 4 shows that AV-SLP_HPV_ vaccination alone does not suffice to permanently eradicate most TC-1 tumors. However, we saw highly potent effects of AV-SLP vaccination in combination treatment with cisplatin and photodynamic therapy (PDT), mediating cures in the large majority of mice where either treatment alone was only effective in a small minority of animals (Fig. 5b, c). This synergistic effect may be explained by several simultaneously operating mechanisms: 1) induction of immunogenic cell death by cisplatin, leading to release of several immunomodulators from dying tumor cells [31]; 2) secretion of TNFα by the SLP vaccine-induced T cells in the tumor microenvironment and increased sensitivity of tumor cells to TNFα-mediated apoptosis in the presence of cisplatin [18], 3) selective depletion of immunosuppressive immature myeloid cells by chemotherapy as was shown for carboplatin and paclitaxel [32]. As the AV-SLP_HPV_ conjugate even more adequately induces T cell responses than SLPs alone, it is likely that the same mechanisms apply here.

Our group recently reported on the synergistic effects of a combination of PDT and immunotherapy [17]. Also, the combination of PDT with AV-SLP_HPV_ vaccination resulted in strongly improved established tumor eradication. The mechanisms underlying the synergism observed by combination of PDT and AV-SLP_HPV_ conjugate vaccination are likely explained by induction of immunogenic cell death [17]. Elevated cross-presentation and T cell activation in combination with our optimized conjugate vaccine resulted in effective synergy in immune control of tumors and cure of almost all animals.

Overall, we conclude that the modification of Pam3CSK4 as applied in the TLR2-L Amplivant™ results in enhanced immunological effects which are beneficial in cancer immunotherapy in mice, particularly when used in synthetic long peptide-conjugate vaccines. These promising results pave the way for further clinical testing of AV-SLP conjugates. Currently, a phase I/II clinical trial has started to assess the dosing and safety profile of two HPV16 E6-derived SLPs conjugated to AV.

## Conclusions

The present study provides insight in the enhanced immunological effects of the novel TLR2-L Amplivant™ (AV) in comparison with the reference compound Pam3CSK4. We show that the enhanced activation of DCs in vitro also translates to enhanced endogenous T cell priming in vivo when AV is conjugated to SLP. For the first time, we show that AV-SLP conjugates applied in an established tumor setting induced therapeutic antitumor efficacy, which was stronger than Pam-SLP_HPV_ conjugate vaccination. The synergy between SLP-conjugated AV and each of two different standard anti-cancer therapies shows the strong therapeutic potential of this TLR2-L.

## Additional file

Additional file 1: **Figure S1.** Activation of B3Z hybridoma after co-culture with D1 DCs loaded with 1uM of indicated compounds. Activation determined by spectrophotometry (OD 595 nm). WT BMDC: BMDCs derived from wildtype C57BL/6; TAPKO BMDC: BMDCs derived from TAP-deficient C57BL/6 mice; SIINFEKL: sequence of short ovalbumin-derived CTL epitope. Similar results were obtained in one additional experiment. Significance determined by unpaired t-test: **** *p* < 0.0001. **Figure S2.** Vaccine-induced HPV16 E7-specific T cell activation. C57BL/6 mice were vaccinated three times s.c. with 5 nmole of either AV-SLP_HPV_ or Pam-SLP_HPV_ conjugate (day 0, 14 and 23). Percentage of D^b^-RAHYNIVTF-tetramer^+^ CD8^+^ T cells detected ex vivo in blood on day 29 (A) and in spleen on day 30 (B). Percentages of effector/effector memory T cells detected ex vivo in spleen (day 30) within tetramer^+^ CD8^+^ T cell population, characterized as CD62L^−^ KLRG1^+^ TM^+^ CD8^+^ T cells (C) and CD127^−^ KLRG1^+^ TM^+^ CD8^+^ T cells (D). **Figure S3.** Maturation of murine DCs as determined by secretion of IL-12p40 and expression of activation markers. (A) Concentration of IL-12p40 detected in the supernatant of 24 h stimulated D1 DCs. Expression of CD86 and MHC class II (B) and CD40 (C) on D1 DCs after stimulation for 24 h with non-conjugated Pam3CSK4 or non-conjugated AV. (D) Concentration of IL-12p40 detected in the supernatant of 24 h stimulated BMDCs and (E) expression of CD40 on BMDCs. Graphs are representative of at least 3 comparable experiments. Significance in **Figure S2. **A and D determined by unpaired t-test: * *p* < 0.05, ** *p* < 0.01, *** *p* < 0.001, **** *p* < 0.0001; Significance in Figure S2.E: Wilcoxon matched-pairs signed-rank test * *p* = 0.016. **Figure S4.** AV-SLP_HPV_ and Pam-SLP_HPV_ have similar potency in induction of T cell tumor infiltration and myeloid cell modulation. (A) Gating strategy for identification of inflammatory macrophages (1), tissue-resident macrophages (2), DC-like macrophages (3) and granulocytic myeloid cells (4). (B) Percentages of CD8^+^ T cells (of live cells; 7-AAD^−^) and K^b^-SIINFEKL^+^ CD8^+^ T cells in tumor tissue as determined by flow cytometry. (C) Percentages of described myeloid subpopulations within CD3^−^CD11b^+^ gated live cells from tumor tissue, depicted as ratio calculated by division by the percentage measured in Untreated animals. (D) Expression of CD11c on described myeloid subpopulations, depicted as ratio calculated by division by the MFI measured in Untreated animals. Data from B-D pooled from two independently and identically performed experiments. Significance determined by unpaired t-test: ** *p* < 0.01, **** *p* < 0.0001. **Figure S5.** Therapeutic vaccination by AV-SLP_HPV_ or Pam-SLP_HPV_ conjugate on day 8 after TC-1 tumor challenge. Data pooled from 5 identically performed experiments; 47 mice per group. (A) Tumor volumes (mm^3^) measured in TC-1 tumor-carrying C57BL/6 mice after vaccination with AV-SLP_HPV_ or Pam-SLP_HPV_ conjugate. X-axis indicates days after vaccination; days of measurements in the different experiments were distributed over 2-day intervals to enable combining of data. Non-parametric Wilcoxon test revealed significant difference (* *p* = 0.016) between vaccinated groups. (B) Kaplan-Meier survival plot of C57BL/6 mice carrying a TC-1 tumor vaccinated with AV-SLP_HPV_ or Pam-SLP_HPV_ conjugate. (PDF 189 kb)

## Abbreviations

APC: Antigen presenting cell
AV: Amplivant
CD: Cluster of differentiation
DC: Dendritic cell
ELISA: Enzyme-linked immunosorbent assay
HPV: Human Papilloma Virus
IFN: Interferon
Pam3CSK4: *N*-palmitoyl-S-(2, 3-bis (palmitoyl)-(2RS)-propyl)-(R)-Cys-Ser-Lys-Lys-Lys-Lys
PAMP: Pathogen-associated molecular pattern
PDT: Photodynamic therapy
SLP: Synthetic long peptide
TLR: Toll-like receptor
TNF: Tumor necrosis factor
