# Supplementary figures and images for: Novel TLR2-binding adjuvant induces enhanced T cell responses and tumor eradication
Source: J Immunother Cancer. 2018 Dec 12;6:146. doi: 10.1186/s40425-018-0455-2 (PMC6292168; doi:10.1186/s40425-018-0455-2)

Suppl Figure 1

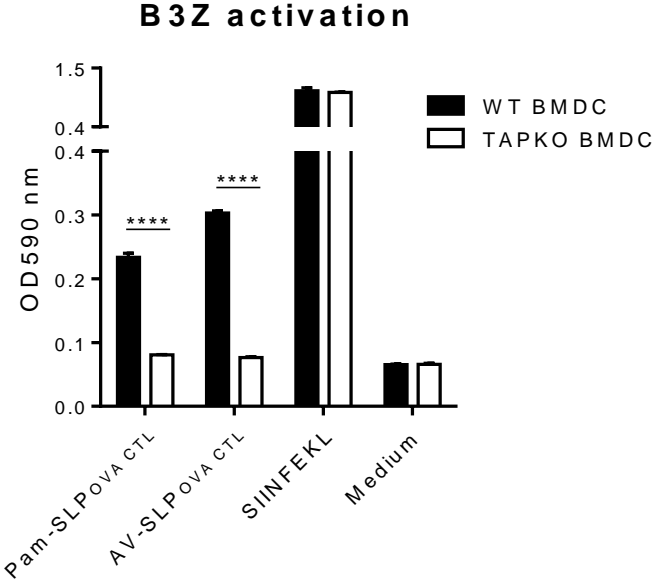

## Suppl Figure 2

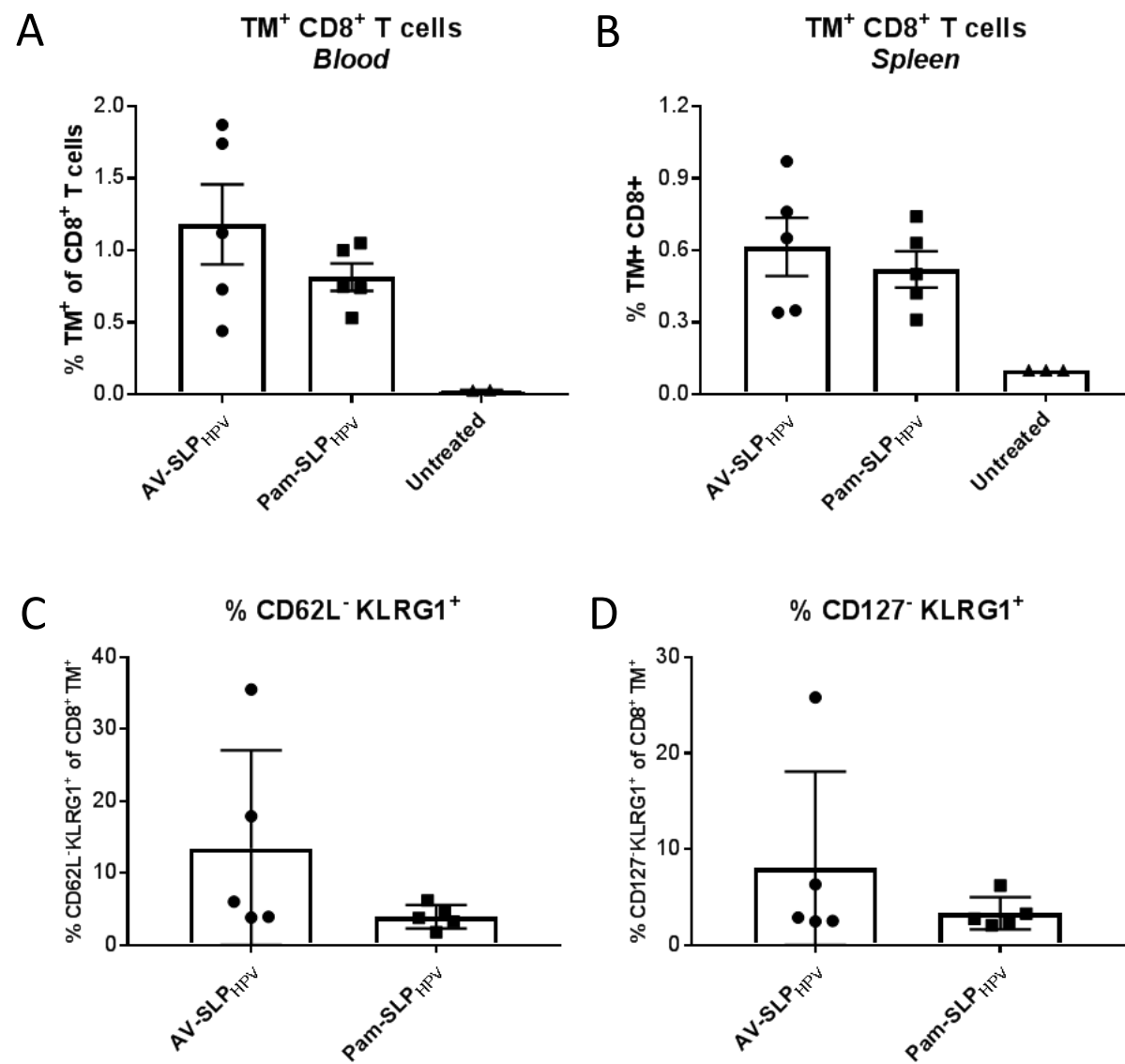

Suppl Figure 3

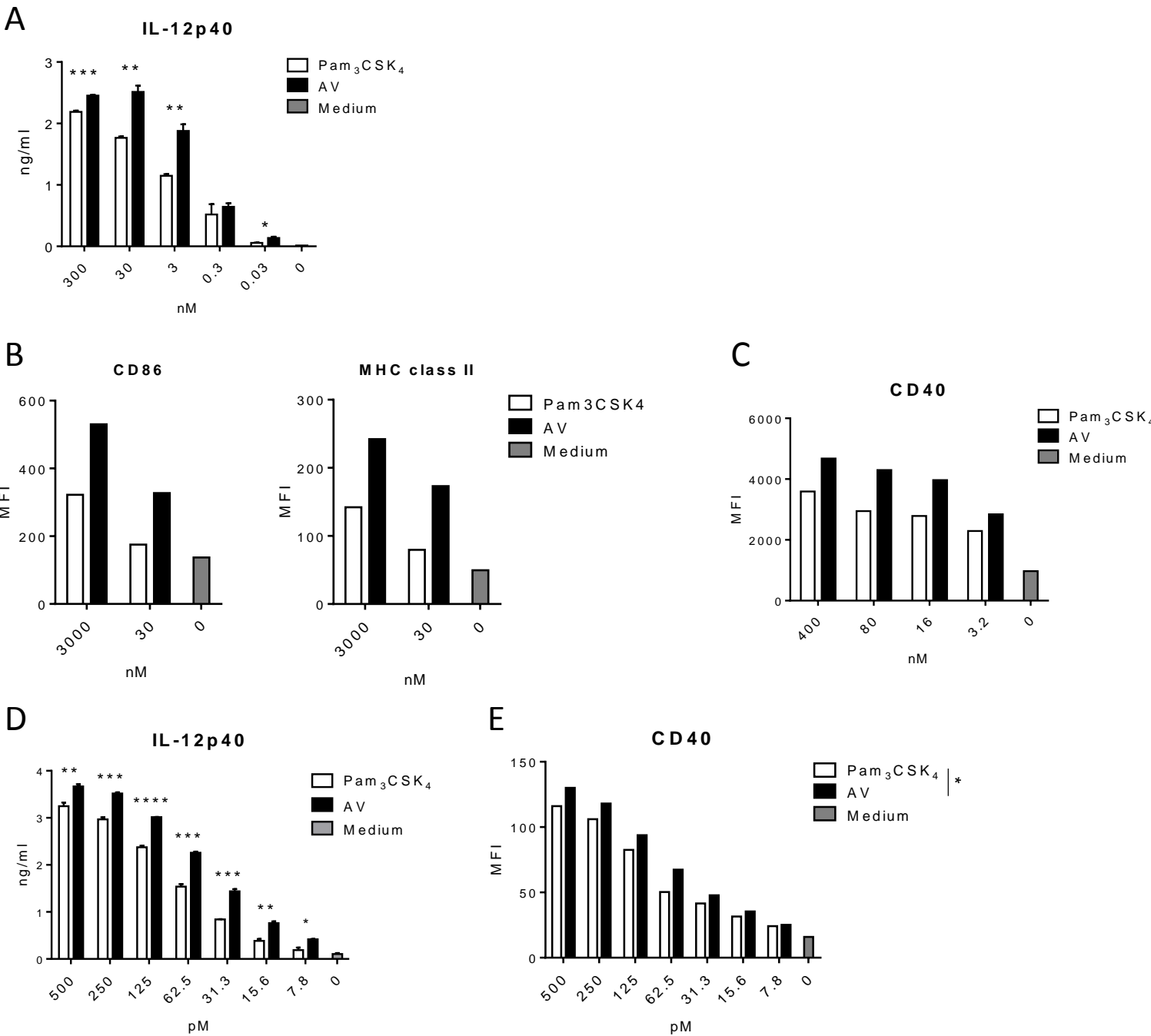

Suppl Figure 4

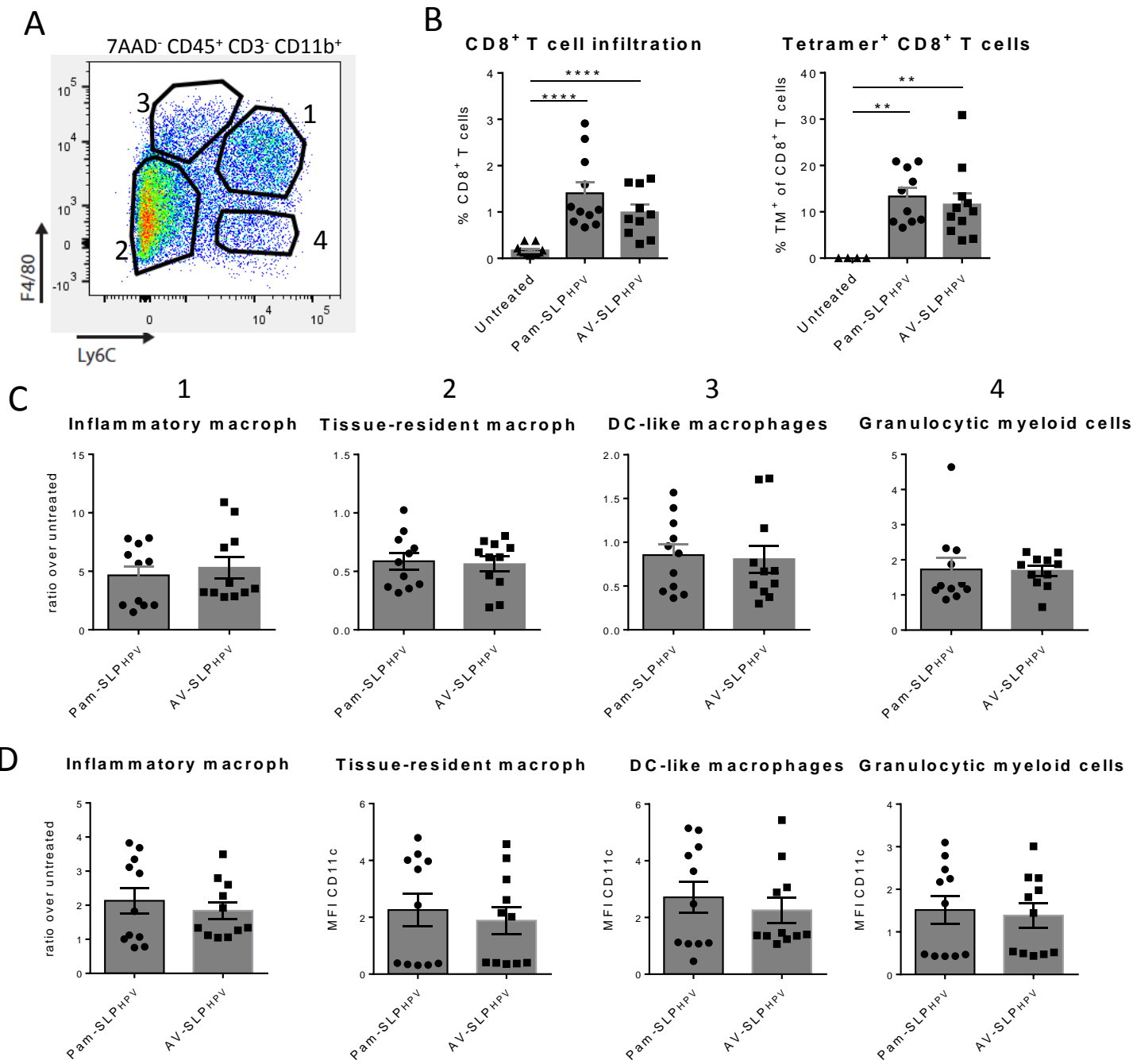

Suppl Figure 5

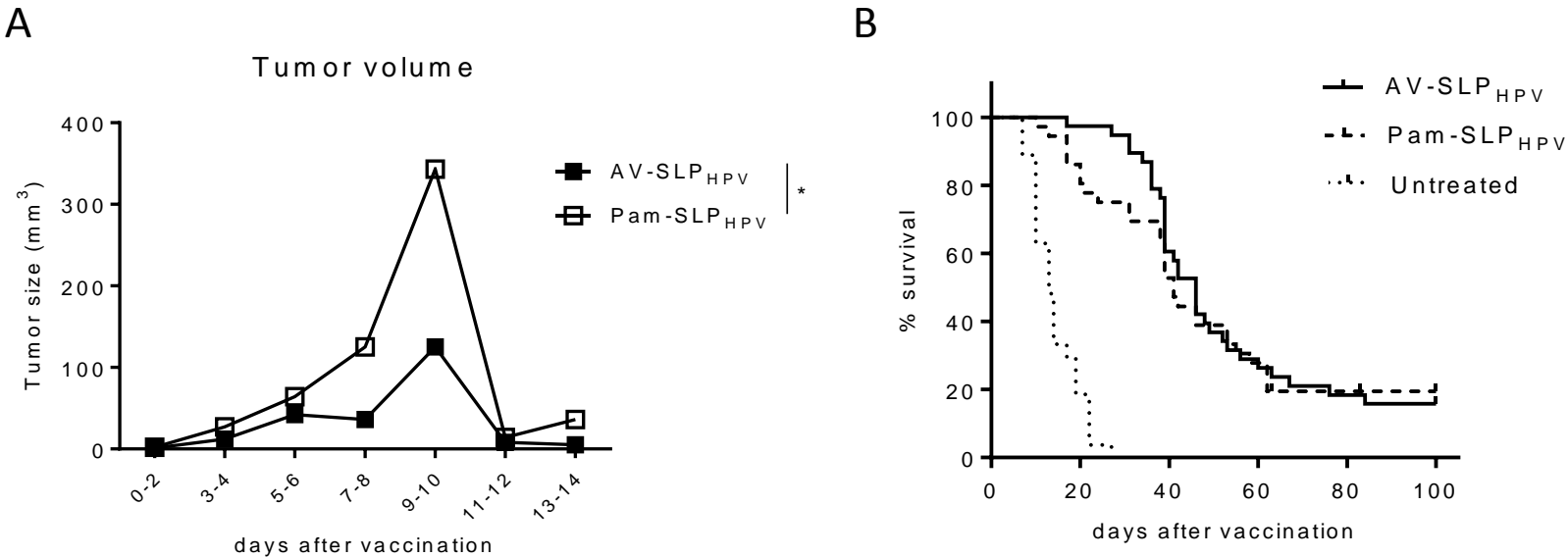

Supplement: Supplementary file 1 — Figure S1. Activation of B3Z hybridoma after co-culture with D1 DCs loaded with 1uM of indicated compounds. Activation determined by spectrophotometry (OD 595 nm). WT BMDC: BMDCs derived from wildtype C57BL/6; TAPKO BMDC: BMDCs derived from TAP-deficient C57BL/6 mice; SIINFEKL: sequence of short ovalbumin-derived CTL epitope. Similar results were obtained in one additional experiment. Significance determined by unpaired t-test: **** p < 0.0001. Figure S2. Vaccine-induced HPV16 E7-specific T cell activation. C57BL/6 mice were vaccinated three times s.c. with 5 nmole of either AV-SLPHPV or Pam-SLPHPV conjugate (day 0, 14 and 23). Percentage of Db-RAHYNIVTF-tetramer+ CD8+ T cells detected ex vivo in blood on day 29 (A) and in spleen on day 30 (B). Percentages of effector/effector memory T cells detected ex vivo in spleen (day 30) within tetramer+ CD8+ T cell population, characterized as CD62L− KLRG1+ TM+ CD8+ T cells (C) and CD127− KLRG1+ TM+ CD8+ T cells (D). Figure S3. Maturation of murine DCs as determined by secretion of IL-12p40 and expression of activation markers. (A) Concentration of IL-12p40 detected in the supernatant of 24 h stimulated D1 DCs. Expression of CD86 and MHC class II (B) and CD40 (C) on D1 DCs after stimulation for 24 h with non-conjugated Pam3CSK4 or non-conjugated AV. (D) Concentration of IL-12p40 detected in the supernatant of 24 h stimulated BMDCs and (E) expression of CD40 on BMDCs. Graphs are representative of at least 3 comparable experiments. Significance in Figure S2. A and D determined by unpaired t-test: * p < 0.05, ** p < 0.01, *** p < 0.001, **** p < 0.0001; Significance in Figure S2.E: Wilcoxon matched-pairs signed-rank test * p = 0.016. Figure S4. AV-SLPHPV and Pam-SLPHPV have similar potency in induction of T cell tumor infiltration and myeloid cell modulation. (A) Gating strategy for identification of inflammatory macrophages (1), tissue-resident macrophages (2), DC-like macrophages (3) and granulocytic myeloid cells (4). (B) Per [file 40425_2018_455_MOESM1_ESM.pdf]
